# Mutations in the *TCAP* gene may lead to restrictive phenotype hypertrophic cardiomyopathy with poor prognosis: case report

**DOI:** 10.1093/ehjcr/ytaf180

**Published:** 2025-04-10

**Authors:** Yunwen Hu, Guangzhong Liu, Jie Yuan, Da Yin, Yaowang Lin

**Affiliations:** Department of Cardiology, Shenzhen People's Hospital (The Second Clinical Medical College, Jinan University, The First Affiliated Hospital, Southern University of Science and Technology), 1017 North Dongmen road, ShenZhen 518020, China; Department of Cardiology, Shenzhen People's Hospital (The Second Clinical Medical College, Jinan University, The First Affiliated Hospital, Southern University of Science and Technology), 1017 North Dongmen road, ShenZhen 518020, China; Cardiovascular Minimally Invasive Medical Engineering Technology Research and Development Center, Shenzhen Key Medical Discipline (SZXK003), 1017 North Dongmen road, Shenzhen 518020, Guangdong, China; Department of Cardiology, Shenzhen People's Hospital (The Second Clinical Medical College, Jinan University, The First Affiliated Hospital, Southern University of Science and Technology), 1017 North Dongmen road, ShenZhen 518020, China; Cardiovascular Minimally Invasive Medical Engineering Technology Research and Development Center, Shenzhen Key Medical Discipline (SZXK003), 1017 North Dongmen road, Shenzhen 518020, Guangdong, China; Department of Cardiology, Shenzhen People's Hospital (The Second Clinical Medical College, Jinan University, The First Affiliated Hospital, Southern University of Science and Technology), 1017 North Dongmen road, ShenZhen 518020, China; Cardiovascular Minimally Invasive Medical Engineering Technology Research and Development Center, Shenzhen Key Medical Discipline (SZXK003), 1017 North Dongmen road, Shenzhen 518020, Guangdong, China; Department of Cardiology, Shenzhen People's Hospital (The Second Clinical Medical College, Jinan University, The First Affiliated Hospital, Southern University of Science and Technology), 1017 North Dongmen road, ShenZhen 518020, China; Cardiovascular Minimally Invasive Medical Engineering Technology Research and Development Center, Shenzhen Key Medical Discipline (SZXK003), 1017 North Dongmen road, Shenzhen 518020, Guangdong, China

**Keywords:** Cardiomyopathy, RP-HCM, TCAP, Heart failure, Case report

## Abstract

**Background:**

Genetic disorders are a significant cause of cardiomyopathies. Mutations in the *TCAP* gene (OMIM #604488) encoding the Z-disc protein Telethonin associated with a mixed phenotype of hypertrophic and restrictive cardiomyopathy with poor prognosis have not yet been reported.

**Case summary:**

A 47-year-old male presented with heart failure symptoms over a year, which had worsened in the past week. He has a familial history of cardiomyopathy, as his mother was diagnosed with restrictive cardiomyopathy (RCM). Transthoracic echocardiography and cardiac magnetic resonance imaging (CMR) revealed non-obstructive hypertrophic cardiomyopathy (HCM) with severe diastolic dysfunction, biatrial enlargement, preserved ejection fraction, and normal chamber size. Endomyocardial biopsy demonstrated cardiomyocyte hypertrophy and focal fibrosis. The patient was diagnosed with hypertrophic cardiomyopathy with a restrictive phenotype (RP-HCM). Whole exome sequencing identified a frameshift *TCAP* mutation producing a truncated product (p.Glu12fs) in the family. Despite interventions, the patient’s cardiac function progressively deteriorated, leading to his placement on the heart transplant waiting list 1 year later.

**Discussion:**

In conclusion, we report for the first time that a heterozygous *TCAP* frameshift mutation resulting in a truncated protein product may contribute to the development of RP-HCM, providing new insights into the genetic basis of cardiomyopathy with mixed phenotypes.

**Learning points**

- The *TCAP* gene mutation (p.Glu12fs) may disrupt myocardial mechano-sensing mechanisms, leading to the rare RP-HCM phenotype.
- The *TCAP* gene mutation (p.Glu12fs) may result in different cardiomyopathy phenotypes and may not exhibit complete penetrance.
- Screening for *TCAP* gene mutations is recommended for patients with RP-HCM and their family members.

## Introduction

Cardiomyopathy encompasses a group of diseases primarily affecting the myocardium, resulting in structural and functional heart abnormalities. Based on clinical features, it can be classified into hypertrophic (HCM), restrictive (RCM), and dilated cardiomyopathy (DCM). The prognosis of cardiomyopathy is primarily determined by its aetiology, with heart failure being a common feature in the end stages.^1^ Inherited cardiomyopathies are caused by genetic mutations, and effective management requires a thorough understanding of the underlying mechanisms. The sarcomere, the smallest contractile unit closely linked to the development of inherited cardiomyopathies, is susceptible to alterations in its functional and structural proteins.^2^ Telethonin, a 19 kDa protein encoded by the *TCAP* gene, plays a crucial role in sarcomere stabilisation by anchoring titin to the Z-disc.^3^ Here, we report a rare cardiomyopathy case with overlapping phenotypes associated with a *TCAP* (OMIM #604488) mutation.

## Summary figure

**Figure:**
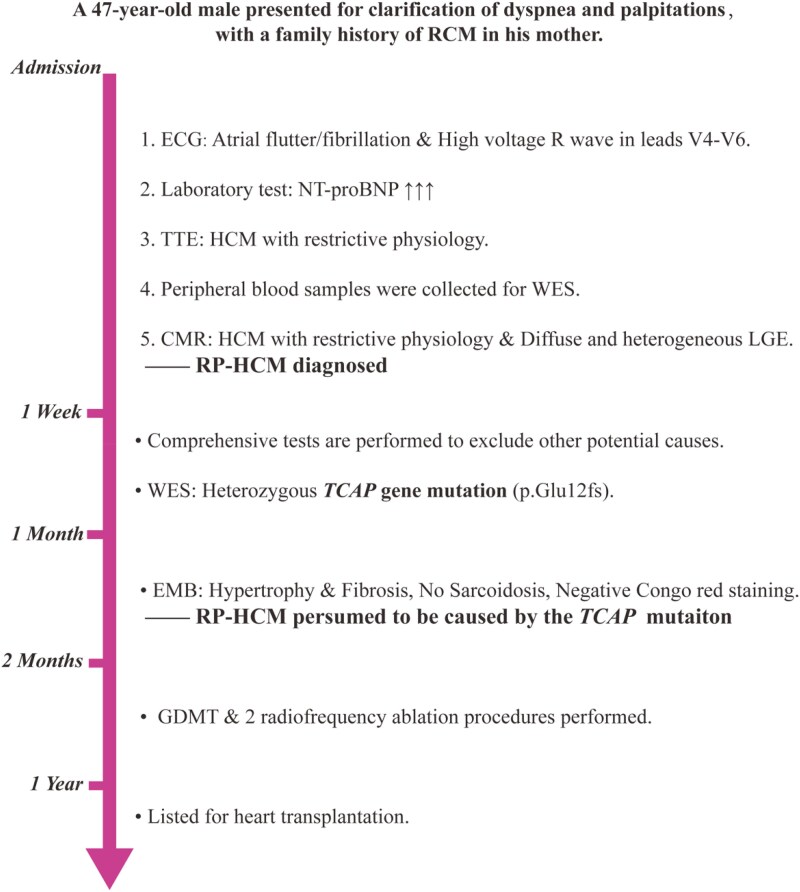


## Case presentation

A 47-year-old man presented with a 1-year history of recurrent exertional chest tightness, shortness of breath, and palpitations, which had worsened over the past week. On examination, he was classified as NYHA class III, with bilateral pedal oedema and bibasilar fine crackles. The patient reported no history of hypertension, diabetes, coronary artery disease, alcohol abuse, or drug use but a family history of cardiomyopathy. Notably, his mother was diagnosed with RCM at the age of 71, 3 years prior, at another institution, where she was identified as a heterozygous carrier of a *TCAP* mutation encoding a truncated protein product (p.Glu12fs). Genetic testing for direct relatives was recommended at that time but not adopted.

**Figure 1:**
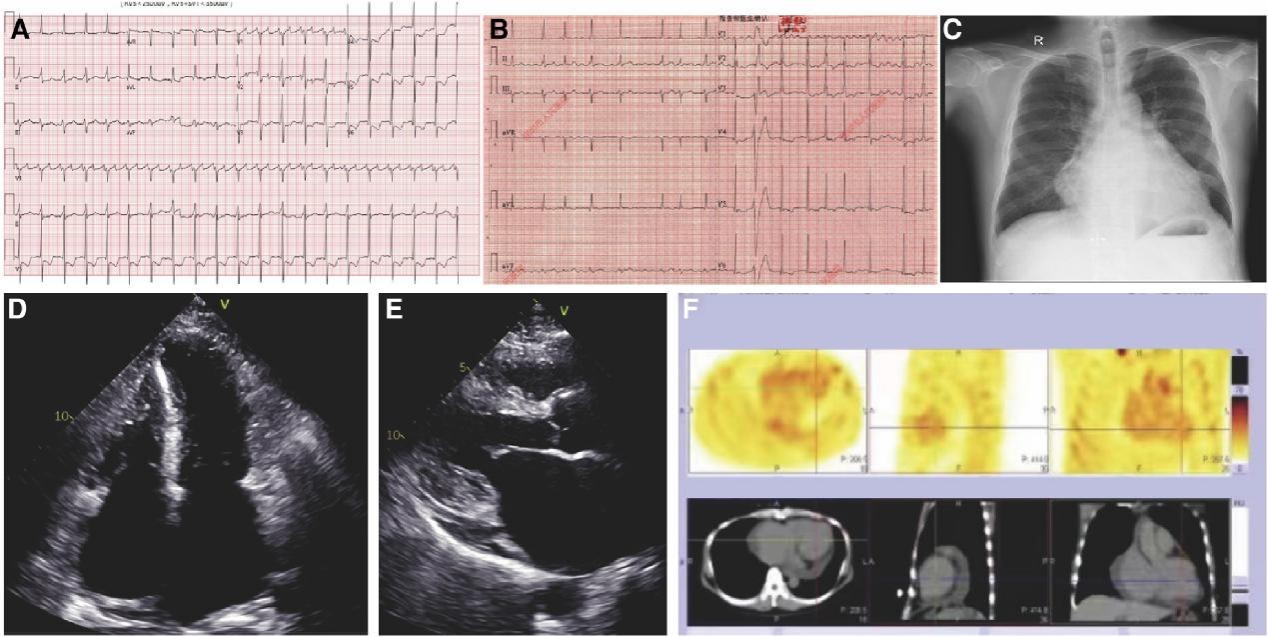
(*A* and *B*) ECG showed atrial flutter and fibrillation with occasional ventricular premature beats. QRS voltage is not reduced. (*C*) Chest X-ray demonstrated an enlarged cardiac silhouette. (*D* and *E*) TTE revealed diffuse wall thickening of the left ventricular wall with coordinated wall motion and no significant regional motion abnormalities. The left ventricle exhibited diastolic dysfunction. The right ventricular apex measured 6 mm thick, and both atria were enlarged. (*F*) The PYP scan showed myocardial uptake comparable to the ribs.

**Figure 2:**
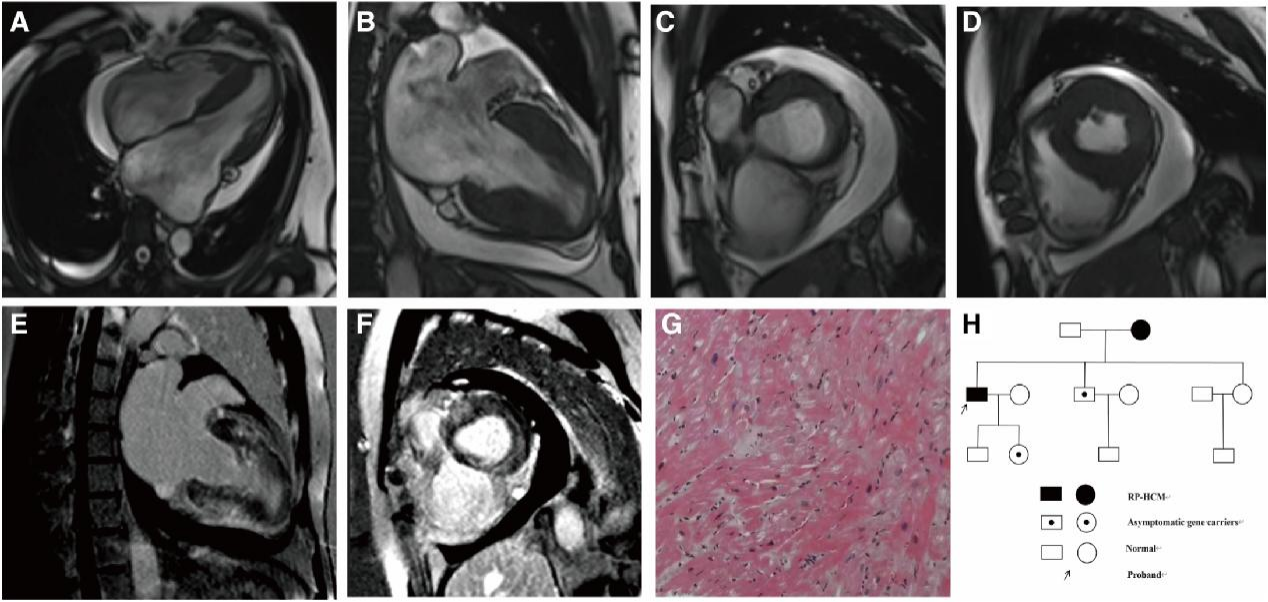
(*A–D*) CMR revealed significant biatrial enlargement. Both ventricles had normal diameters, but diastolic dysfunction was noted. Segmental hypertrophy of the left ventricular wall was observed, with varying degrees of thickening: septum 14–22 mm, anterior wall 10–20 mm, lateral wall 8–14 mm, and inferior wall 7–14 mm. The right ventricular wall was mildly thickened. The left ventricular ejection fraction was 61%, and the end-diastolic volume index was 36.8 mL/m^2^. (*E* and *F*) The LGE revealed extensive intramyocardial enhancement of the septum, as well as the anterior and inferior walls of the left ventricle. (*G*) H&E staining revealed enlarged cardiomyocytes with hyperchromatic nuclei, indicating hypertrophy, along with focal fibrosis. (*H*) Pedigree chart: the proband presented with RP-HCM, the proband's mother was diagnosed with RCM, and the proband's brother and daughter are asymptomatic carriers.

After admission, the electrocardiogram (ECG) revealed atrial flutter/fibrillation and R waves with high voltage in leads V4 to V6 (*Figure 1A* and *B*). Laboratory tests showed normal levels of acute-phase proteins and cardiac enzymes, as well as normal liver and renal function. However, the NT-proBNP level was markedly elevated at 6723 pg/mL (reference range: <125 pg/mL). The transthoracic echocardiogram (TTE) revealed hypertrophy of the lateral wall (14 mm), posterior wall (16 mm), and septum (14.5 mm), along with biatrial enlargement. Severe left ventricular diastolic dysfunction was also noted (E/lateral e′ = 13.9, E/septal e′ = 18.1), although the left ventricular ejection fraction was preserved at 65% (*Figure 1D* and *E*, Supplementary material online). Tc99m-pyrophosphate (PYP) scan showed Perugini scores of 2 and 1 at 1 and 3 h post radiotracer infusion (*Figure 1F*). Cardiac magnetic resonance imaging (CMR) confirmed concentric left ventricular wall hypertrophy with reduced myocardial motion and diffuse heterogeneous late gadolinium enhancement (LGE) (*Figure 2A–F*). No evidence of left ventricular outflow tract obstruction or aortic stenosis was identified by either TTE or CMR. Coronary angiography revealed no evidence of coronary artery stenosis. Endomyocardial biopsy (EMB) showed hypertrophy of cardiomyocytes, along with focal fibrosis (*Figure 2G*). Given the different cardiac phenotype compared with his mother, whole exome sequencing (WES) was performed, which revealed that the patient had a *TCAP* mutation encoding the same defective protein as that carried by his mother (p.Glu12fs). Meanwhile, his daughter and brother were found to carry the *TCAP* gene mutation, but both presented with normal phenotypes (*Figure 2H*).

The patient was ultimately diagnosed with HCM with restrictive phenotype (RP-HCM), which was presumed to be caused by the *TCAP* mutation. He received diuretic therapy and standard heart failure treatment, including Dapagliflozin (10 mg qd), Metoprolol (23.75 mg qd), Sacubitril Valsartan Sodium (24/26 mg bid), and Spironolactone (20 mg qd). Rivaroxaban (20 mg qd) was administered for thromboprophylaxis, and radiofrequency ablation was performed to manage atrial arrhythmias. Despite these interventions, over the course of a year, the patient experienced multiple unplanned visits, two re-hospitalisations for heart failure, and underwent a successful second radiofrequency ablation for recurrent atrial fibrillation. Unfortunately, atrial fibrillation recurred shortly after the procedure, and the patient continued to experience recurrent heart failure exacerbations, requiring additional unplanned visits. Ultimately, the patient was listed for heart transplantation.

## Discussion

This is the first report of an RP-HCM case associated with a heterozygous *TCAP* frameshift mutation, resulting in a truncated protein that replaces the C-terminal 159 amino acids with 19 aberrant amino acids. Previously, this genetic defect had been identified only in families with homozygous limb-girdle muscular dystrophy type 2G^4,5^ RP-HCM with severe diastolic dysfunction is relatively rare, accounting for <3% of hypertrophic cardiomyopathy (HCM) cases^6,7^ The patient’s family history raised the suspicion of genetic cardiomyopathy. However, given the absence of prior reports linking *TCAP* mutations to RCM or RP-HCM, and in consideration of the patient’s preferences, WES was performed. Concurrently, laboratory tests, imaging tests, and EMB were conducted. Of note, the patient’s PYP scan yielded ambiguous results. However, the PYP scan is not specific for amyloidosis, and a false-positive result with a Perugini score below three has been reported in a case of inherited cardiomyopathy caused by *MYBPC3* mutation.^8^ Amyloidosis was then excluded based on high ECG voltages, normal serum free light chain levels, normal immunofixation electrophoresis results, and negative Congo red staining (not shown). Fabry disease was unlikely due to normal α-galactosidase A activity, and cardiac sarcoidosis was excluded based on EMB findings. Ultimately, the patient’s RP-HCM was attributed to the *TCAP* mutation. Notably, the patient also exhibited diastolic dysfunction in the right ventricle, with hypertrophy of the right ventricular apex, suggesting right ventricular involvement. However, the presence of moderate pericardial effusion and elevated pulmonary circulation pressure (pulmonary artery systolic pressure: 49 mmHg), secondary to severe left ventricle diastolic dysfunction, complicates the interpretation.

This patient has a family history of RCM, and according to the diagnostic criteria proposed by Kubo *et al*.^7^, the diagnosis of RP-HCM requires a first-degree relative with a history of HCM.^7^ However, HCM and primary RCM may represent different phenotypes of the same disease, termed ‘sarcomere cardiomyopathy’. Furthermore, studies suggest that RP-HCM patients have a poor prognosis, similar to that of primary RCM.^6,7,9^ This indicates that diastolic dysfunction could be the primary factor contributing to the poor prognosis of these patients. Based on the present case, it could be proposed that the diagnosis of RP-HCM may not always require a confirmed HCM diagnosis in a first-degree relative, as the patient’s cardiac morphology and functional characteristics appear to be adequate for prognosis assessment. Nevertheless, genetic testing remains necessary, not only to provide genetic counselling and early screening for the patient’s family, but also to identify rare genetic cardiomyopathies and exclude other inherited disease subtypes.

Although rare, *TCAP* mutations have been reported in several families with HCM and DCM.^10,11^ The specific phenotype of cardiomyopathy associated with *TCAP* mutations may depend on how these mutations affect the interaction between telethonin and other Z-disc proteins such as titin, calsarcin, and muscle LIM protein (MLP). A Y2H assay showed that *TCAP* mutations from DCM families impair the interaction between telethonin and Z-disc proteins while those from HCM families enhance these interactions.^10^ However, telethonin may not be essential for titin anchoring in mammals. In vivo experiments indicate that titin can maintain its connection to the Z-disc via actin despite complete *TCAP* knockout, and the DCM phenotype manifests only when interactions among titin, actin, and telethonin are simultaneously disrupted.^10,12,13^ In our case, although the *TCAP* mutation disrupted all critical interaction sites with titin, calsarcin, and MLP, the patient presented with RP-HCM rather than typical DCM or HCM. We hypothesize this may be attributed to impaired mechano-transcriptional coupling in cardiomyocytes. Compared with his mother (1.56 m, 50 kg, BMI 20.5 kg/m^2^), the patient performs more cardiac work due to being overweight (1.63 m, 75 kg, BMI 28.3 kg/m^2^) and engaging in more strenuous physical labor. Studies have shown that *TCAP*-deficient mice subjected to increased myocardial biomechanical load exhibit elevated myocardial ROS levels, enhanced autophagy, and fibrosis, leading to rapid maladaptive hypertrophy and heart failure.^14,15^ Additionally, his 20-year history of smoking may have contributed to increased cardiomyocyte oxidative stress. The patient's 40-year-old brother and 16-year-old daughter, both carriers of the *TCAP* mutation, did not manifest clinical symptoms, potentially because their accumulated myocardial workload had not yet reached the pathogenic threshold.

In conclusion, we report a rare case of RP-HCM potentially caused by a *TCAP* gene mutation resulting in a truncated protein product (p.Glu12fs). This case highlights the need for awareness of poor prognosis in patients with a mixed phenotype characterized by both myocardial hypertrophy and significant diastolic dysfunction, and that the diagnosis of RP-HCM might not necessarily require a confirmed family history of HCM. Furthermore, *TCAP* gene mutations may lead to various cardiomyopathy phenotypes, making genetic screening for *TCAP* mutations clinically significant. However, the mechanisms through which *TCAP* mutations cause cardiomyopathy remain unclear, and further research is needed.

## Supplementary Material

ytaf180_Supplementary_Data

## Data Availability

The data underlying this article will be shared on reasonable request to the corresponding author.

## Lead author biography

Dr Yaowang Lin is working as a cardiologist in the Department of Cardiology, Shenzhen People's Hospital. He specializes in internal medicine and cardiovascular disease, with a particular focus on the diagnosis and interventional treatment of heart failure, cardiomyopathies, and structural heart diseases.

## Notes

**Consent:** In accordance with the COPE guidelines, written informed consent was obtained from the patient for the publication of this case report, including all associated images and data.

**Funding**: This study is supported by Shenzhen Science and Technology Program (NO. JCYJ20240813104359020).
