# Supplementary figures and images for: Mutations in the TCAP gene may lead to restrictive phenotype hypertrophic cardiomyopathy with poor prognosis: case report
Source: Eur Heart J Case Rep. 2025 Apr 10;9(5):ytaf180. doi: 10.1093/ehjcr/ytaf180 (PMC12053251; doi:10.1093/ehjcr/ytaf180)

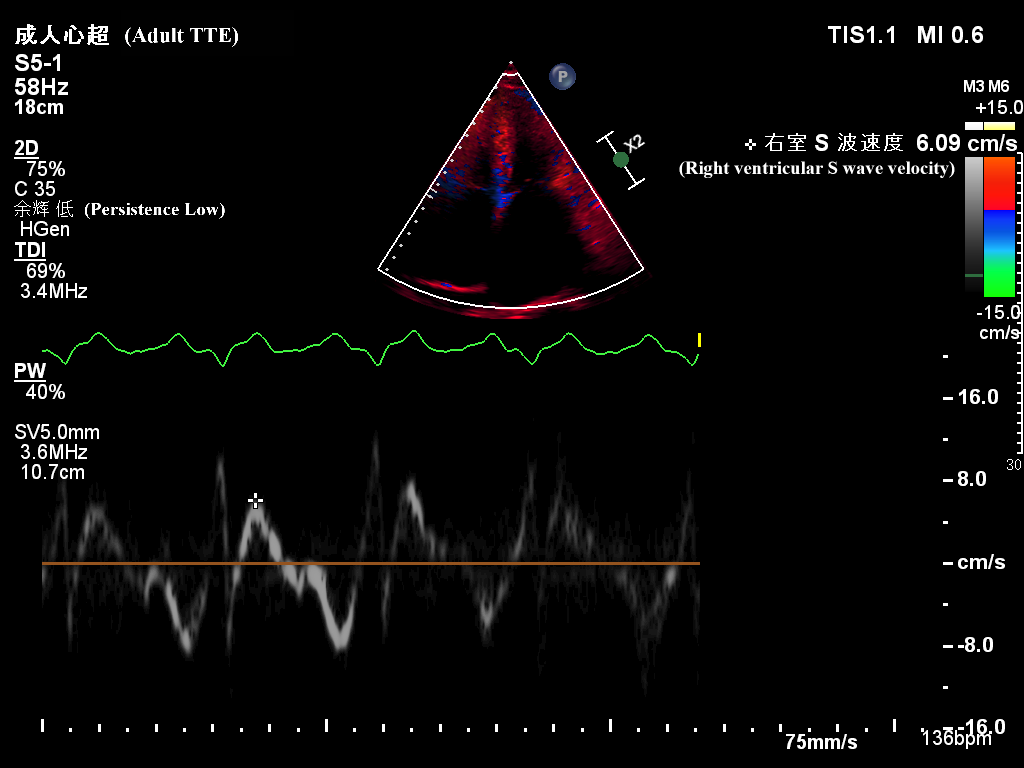

Supplement: ytaf180_Supplementary_Data [file ytaf180_supplementary_data.zip › Supplemental figure S1.Jpg]

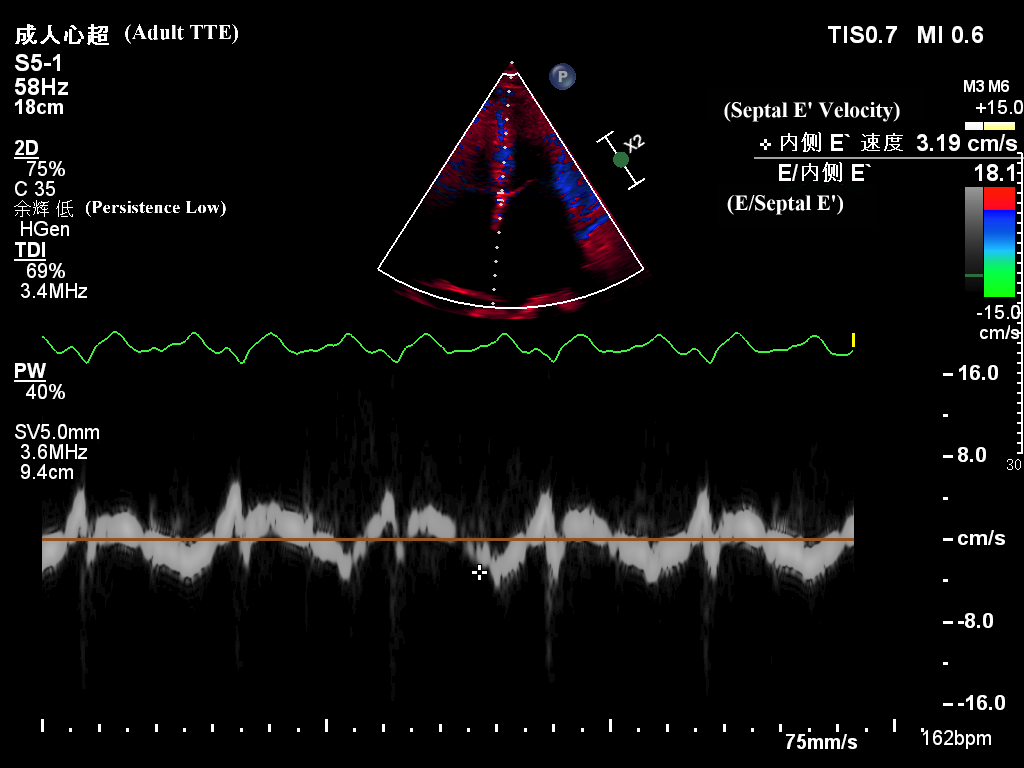

Supplement: ytaf180_Supplementary_Data [file ytaf180_supplementary_data.zip › Supplemental figure S2.Jpg]

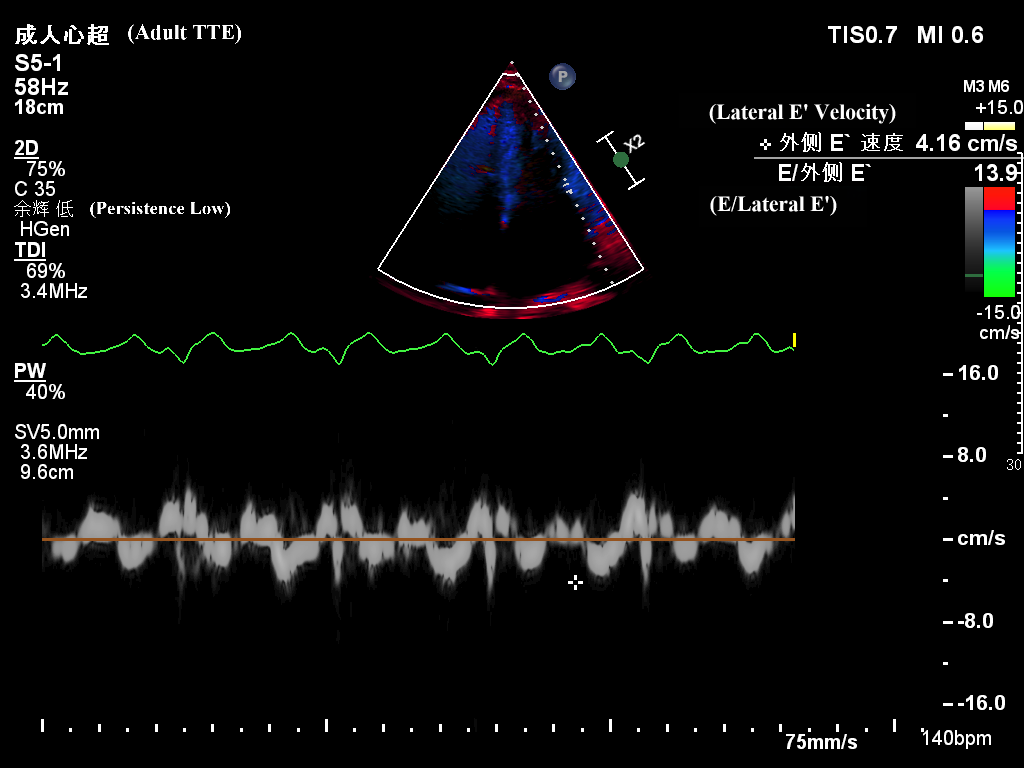

Supplement: ytaf180_Supplementary_Data [file ytaf180_supplementary_data.zip › Supplemental figure S3.Jpg]
